# Cortical Thickness Predicts Response Following 2 Weeks of SSRI Regimen in First-Episode, Drug-Naive Major Depressive Disorder: An MRI Study

**DOI:** 10.3389/fpsyt.2021.751756

**Published:** 2022-02-22

**Authors:** Peiyi Wu, Aixia Zhang, Ning Sun, Lei Lei, Penghong Liu, Yikun Wang, Hejun Li, Chunxia Yang, Kerang Zhang

**Affiliations:** ^1^Department of Psychiatry, First Hospital of Shanxi Medical University, Taiyuan, China; ^2^Department of Psychiatry, Shanxi Medical University, Taiyuan, China; ^3^Department of Mental Health, Shanxi Medical University, Taiyuan, China

**Keywords:** major depression disorder (MDD), cortical thickness, selective serotonergic reuptake inhibitors (SSRI), MRI, predict

## Abstract

**Objective:**

Major depression disorder (MDD) is a harmful disorder, and the pathological mechanism remains unclear. The primary pharmacotherapy regimen for MDD is selective serotonin reuptake inhibitors (SSRIs), but fewer than 40% of patients with MDD are in remission following initial treatment. Neuroimaging biomarkers of treatment efficacy can be used to guide personalized treatment in MDD. This study aims to determine if cortical thickness can be used as a predictor for SSRIs.

**Methods:**

A total of 126 first-episode, drug-naive MDD patients (MDDs) and 71 healthy controls (HCs) were enrolled in our study. Demographic data were collected according to the self-made case report form (CRF) at the baseline of all subjects. Magnetic resonance imaging (MRI) scanning was performed for all the participants at baseline, and all imaging was processed using the DPABISurf software. All MDDs were treated with SSRIs, and symptoms were assessed at both the baseline and 2 weeks using the 17-item Hamilton Rating Scale (HAMD-17). According to HAMD-17 total score improvement from baseline to the end of 2 weeks, the MDDs were divided into the non-responder group (defined as ≤ 20% HAMD-17 reduction) and responder group (defined as ≥50% HAMD-17 reduction). The receiver operating characteristic (ROC) curve was used to analyze the diagnostic value of MDDs' and HCs' cortical thickness for MDD. Correlation analysis was performed for the responder group and the non-responder group separately to identify the relationship between cortical thickness and SSRI treatment efficacy. To analyze whether cortical thickness was sufficient to differentiate responders and non-responders at baseline, we used ROC curve analysis.

**Results:**

Significant decreases were found in the cortical thickness of the right supplementary motor area (SMA) in MDDs at the baseline (corrected by the Monte Carlo permutation correction, cluster-wise significant threshold at *p* < 0.025 and vertex-wise threshold at *p* = 0.001), area under the curve (AUC) = 0.732 [95% confidence interval (CI) = 0.233–0.399]. In the responder group, the cortical thickness of the right SMA was significantly thinner than in the non-responder group at baseline. There was a negative correlation (*r* = −0.373, *p* = 0.044) between the cortical thickness of SMA (0 weeks) and HAMD-17 reductive rate (2 weeks) in the responder group. The results of ROC curve analyses of the responder and non-responder groups were AUC = 0.885 (95% CI = 0.803–0.968), sensitivity = 73.5%, and specificity = 96.6%, and the cutoff value was 0.701.

**Conclusion:**

Lower cortical thickness of the right SMA in MDD patients at the baseline may be a neuroimaging biomarker for MDD diagnosis, and a greater extent of thinner cortical thickness in the right SMA at baseline may predict improved SSRI treatment response. Our study shows the potential of cortical thickness as a possible biomarker that predicts a patient's clinical treatment response to SSRIs in MDD.

## Introduction

Major depression disorder (MDD) is a common, costly, and disabling mental disorder with poor health outcomes (1, 2). Selective serotonin reuptake inhibitors (SSRIs) are a second-generation antidepressant most often used and currently recommended as the first-line therapy for MDD patients (3–5). However, because of the unclear pathogenesis of MDD, fewer than 40% of patients with MDD go into remission after initial treatment (6, 7). Moreover, 70% of MDD patients need several sessions of treatment or even consecutive treatment trials to experience remission (8), which further delays patients' recovery. Delays reduce patient compliance and potentially increase the risk of suicide. A large number of studies show that improvement in the first 2 weeks with SSRIs may effectively predict the following treatment outcome and that a lack of symptomatic improvement in the first 2 weeks may be a more robust predictor of non-remission after 6–8 weeks (9–20). In terms of clinical treatment decision-making, among patients with MDD who demonstrate no improvement in the first 2 weeks of treatment and continue the initial strategy, only 4% could become stable responders. The above results indicate that the treatment strategy needs to be changed after 2 weeks of treatment failure (9, 21). Thus, the identification of biomarkers to guide MDD treatment may improve the MDD clinical outcomes. To date, however, no clinically useful biomarker for MDD treatment response has emerged.

Structural magnetic resonance imaging (sMRI) research can offer important insights into brain changes associated with MDD, and it has become a promising method for finding biomarkers of MDD. One sMRI parameter is cortical thickness, which is a collection of brain subtle features; this is associated with multifaceted advanced cognitive functions and can be seen as a relatively reliable and stable structural brain feature throughout the human life span (22–24). It seems to be that any subtle brain structural features could predict the degree of short-term response to drug therapy (25), and cortical thickness is more sensitive to disease-related gray matter changes than voxel-based morphometry (26, 27). Previous research has shown that abnormal cortical thickness in early life stages is an important risk factor for MDD (28). Cortical thickness changes have also been confirmed to be presented in MDD and have significant associations with clinical measures in MDD (29–33). Moreover, cortical thickness has been shown in many research studies to predict the electroconvulsive therapy (ECT) clinical efficacy (34–36). As for drug treatment, Bartlett et al. found that in the first week of treatment with sertraline, cortical thickness changes in the bilateral anterior cingulate were associated with the final clinical outcome of MDD (30). However, few studies were conducted to examine cortical thickness as an indicator to predict exclusively the SSRI treatment efficacy in first-episode, drug-naive MDD patients. To fill this void, we performed this sMRI study.

In the present study, we used MRI technology to investigate the cortical thickness of patients with MDD at baseline and to investigate if and to what extent cortical thickness as biomarker can predict the therapeutic efficacy of SSRIs. Identification of such biomarkers for MDD could aid in clinical decision making and individualized treatment and decrease the cost burden on patients, thereby ensuring that patients have a good quality of life.

## Methods

### Participants

In this study, 126 cases of first-episode, drug-naive MDD patients were all enrolled from the Department of Psychiatry of the First Hospital of Shanxi Medical University from September 2010 to December 2019. Seventy-one matched healthy controls (HCs) were likewise enrolled from local universities and local communities. All the participants were from the Han Chinese population. After we explained the study protocol, all subjects agreed to participate in the study and provided signed and written informed consent. This study was approved by the ethical committee of the First Hospital of Shanxi Medical University.

Patients with MDD were screened by at least two experienced psychiatrists using the Chinese version of the Structured Clinical Interview for the *Diagnostic and Statistical Manual of Mental Disorders, Fourth Edition* (*DSM-IV*) (SCID-I/P). The diagnosis of MDD was made according to the “mood episodes” section in *DSM-IV*, and patients were required to meet the conditions of single-episode MDD. The entry criteria of MDDs were as follows: aged 18–55 years; right-handed; meet the *DSM-IV* criteria for single-episode MDD; first-onset, drug-naive; 17-item Hamilton Depression Rating Scale (HAMD-17) score >17; 14-item Hamilton Anxiety Rating Scale (HAMA-14) score <14; no comorbid Axis I psychiatric disorders; and no history of serious suicide, self-harm, or active suicidal ideation. Healthy control inclusion criteria were as follows: aged 18–55 years; right-handed; not meet the *DSM-IV* diagnostic criteria for a single episode of major depressive disorder and any mental diagnosis criteria of disorders; and no history of any mental disorders. Exclusion criteria for all the participants were as follows: severe organic diseases (neurological disorder, head injury, liver/kidney function abnormalities, cardiovascular abnormalities, etc.), family history of psychotic disorder, abuse of alcohol or substances, MRI contraindications (e.g., claustrophobia, metallic implants), and pregnant or lactating women.

### Clinical Assessment and Treatment Protocol

All participants performed clinical assessment and received an MRI scan at baseline, and then MDD patients consented to commence SSRIs as treatment as usual at a low dose. Afterward, the dose for each individual was adjusted according to each patient's response and tolerance. Selective serotonin reuptake inhibitor dosages for antidepressant treatments in MDD group were as follows: 20–50 mg/day for fluoxetine, 20–50 mg/day for paroxetine, 20–60 mg/day for citalopram, 50–200 mg/day for sertraline, and 5–20 mg/day for escitalopram. Patients with insomnia were combined with benzodiazepines or supportive psychotherapy for a short time. Other antidepressants, anxiolytics, antipsychotics, electroconvulsions, or other physical therapy were not combined within 2 weeks. Two weeks following SSRI treatment, MDDs were assessed with HAMD-17.

Our study evaluated the therapeutic effects of SSRIs based on the reduction rate of HAMD-17 after 2 weeks of treatment. Responders were defined as HAMD-17 scores reduced by more than 50% after 2 weeks of SSRI treatment, and the patients with MDD showed a clinical improvement after SSRI treatment. Non-responders were defined as <20% reduction in HAMD-17 scores.

### MRI Data Acquisitions

Using the 3T Siemens Trio (A Tim System) full-body scanner at the First Hospital of Shanxi Medical University, sMRI data were acquired at the baseline; T1-weighted (T1w) MRI scans were obtained by sagittal three-dimensional fast low-angle shot sequence using the following parameters: repetition time = 2,300 ms, echo time = 2.95 ms; inversion time (TI) = 900 ms; flip angle (FA) = 9°; acquisition matrix = 256 × 240; number of slices = 160; field of view (FOV) = 225 × 240 mm^2^; thickness = 1.2 mm, gap = 0.6 mm; time = 9 min 14 s.

All participants were trained and pretested before undergoing MRI scan, and they were informed of the inspection procedures, approximate time required, and possible adverse effects. During the scan, subjects remained awake with their eyes closed, and we provided participants with headphones to lessen the noise from MRI machine. To minimize the effect of head movement, we placed sponge pads in the coil and told participants not to move their heads.

### Imaging Processing for Cortical Thickness Analysis

For the whole-brain cortical thickness analysis, we used DPABISurf_V1.2 (http://rfmri.org/DPABISurf) (31) to process and analyze data through a series of steps. DPABISurf is a surface-based resting-state functional MRI data analysis toolbox based on fMRIPrep 20.2.1 (37) RRID:SCR_016216 and FreeSurfer 6.0.1 (38). The T1w images were used to calculate the cortical thickness of the whole brain. The specific image-processing procedures were as follows: the T1w image was corrected for intensity non-uniformity with N4BiasFieldCorrection (39), distributed with ANTs 2.2.0 (40), and used as a T1w reference throughout the workflow. The T1w reference was then skull-stripped with a Nipype implementation of the antsBrainExtraction.sh workflow (ANTs2.2.0), using OASIS30ANTs as the target template. Brain tissue segmentation of cerebrospinal fluid, white-matter, and gray matter was performed on the brain-extracted T1w using fast (FSL 5.0.9) (41). Brain surfaces were reconstructed using recon-all (Free Surfer 6.0.1) (38), and the brain mask estimated previously was refined with a custom variation of the method to reconcile ANTs-derived and FreeSurfer-derived segmentations of the cortical gray matter of Mindboggle (42). Volume-based spatial normalization to one standard space (MNI152NLin2009cAsym) was performed through non-linear registration with antsRegistration (ANTs 2.2.0), using brain-extracted versions of both the T1w reference and the T1w template. The following template was selected for spatial normalization: ICBM 152 Nonlinear Asymmetrical template version 2009c (43).

Participants who had any head shift >1.5 mm or rotation >1.5° were excluded from the analyses.

### Statistical Analysis

All the clinical data and demographic data analysis were performed using SPSS 22.0. For the imaging analysis, after the exclusion of ineligible sMRI data, in total, 100 MDD patients and 52 HCs were included in this study. The cortical thickness was used as the index, and the difference between the cortical thickness of the MDD group and the HC group at baseline was compared by independent-sample *t*-test (age, sex, education, and FD Jenkinson were regressed in the independent-sample *t*-test to avoid their influence), and the different brain areas were extracted. For multiple comparisons correction, we used Monte Carlo Null-Z simulation with 10,000 permutations (two tailed). The cluster-wise significant threshold was set at *p* < 0.025 (each hemisphere), and the vertex-wise threshold was set at *p* = 0.001. DPABISurf provided an index table, which listed different *p*-values of various cluster sizes corresponding to its smoothing levels. Finally, the receiver operating characteristic (ROC) curve was applied for analyzing the diagnostic value of the difference in cortical thickness between MDDs and HCs for MDD.

According to the HAMD-17 total score improvement from baseline to the end of 2 weeks ≥50% or ≤ 20%, all the baseline MDDs were divided into the responder group (*n* = 29) and non-responder group (*n* = 34). The different cortical thickness regions (after correction) between MDDs and HCs were used as mask; then, an independent *t*-test was used to compare the different cortical thickness between the responder group and the non-responder group of SSRI drugs. The average cortical thickness of each significant region was extracted for further group-level analysis, and then ROC curve analysis was performed on the responder group and the non-responder group.

We extracted the average cortical thickness differences between the responder group and the non-responder group at baseline and then performed partial correlation analysis for measuring the correlation between the averaged cortical thickness at baseline and the HAMD-17 score improvement from baseline (sex, age, and education were taken as covariables).

## Results

### Demographic and Clinical Comparisons

The demographic data comparisons between the (1) MDDs and HCs and the (2) responder group and the non-responder group are presented in Tables 1, 2, respectively. All the intergroup comparisons were not statistically significant in age, sex, and education. HAMD-17 and HAMA-14 scores have no statistical differences between the two groups at baseline.

**Table 1:** The demographic and clinical data between the MDD group and the HC group at baseline.

**Variable**	**MDD** **(*n* = 100)**	**HC** **(*n* = 52)**	***p***
Age (years)	33.97 ± 9.50	36.71 ± 8.18	0.079^a^
Sex (M/F)	43/57	29/23	0.135^b^
Education	4.56 ± 1.29	4.79 ± 0.97	0.264^a^
HAMD-17 (0 week)	21.70 ± 2.72	—	—
HAMA-14 (0 week)	10.60 ± 1.77	—	—

*Education: 1, illiterate; 2, primary school; 3, lower secondary school; 4, high school; 5, college; 6, university; 7, graduate or greater. HAMD-17, 17-item Hamilton Depression Rating Scale*.

a *Independent-sample t-test*.

b *χ^2^-test*.

**Table 2:** The demographic and clinical data between responders group and non-responder group at baseline and 2 weeks.

**Variable**	**Responder group** **(*n* = 29)**	**Non-responder group** **(*n* = 34)**	***p***
Age (years)	33.14 ± 9.62	33.12 ± 10.21	0.994^a^
Sex (M/F)	11/18	14/20	0.793^b^
Education (years)	4.24 ± 1.32	4.74 ± 1.28	0.140^a^
HAMD-17 (0 week)	22 ± 3.05	21.94 ± 2.91	0.938^a^
HAMD-17 (2 week)	10.14 ± 1.70	18.68 ± 2.37	<0.001^a^
HAMD-17 reductive rate	0.54 ± 0.03	0.14 ± 0.05	<0.001^a^
HAMD-17 reduction	11.86 ± 1.73	3.26 ± 1.24	<0.001^a^
HAMA-14 (0 week)	10.50 ± 1.77	10.72 ± 1.79	0.621^a^
HAMA-14 (2 week)	9.793 ± 1.52	10.17 ± 1.99	0.400^a^

*Education: 1, illiterate; 2, primary school; 3, lower secondary school; 4, high school; 5, college; 6, university; 7, graduate or greater. HAMD-17, 17-item Hamilton Depression Rating Scale*.

a *Independent-sample t-test*.

b χ*^2^-test*.

After 2 weeks of SSRI treatment, the HAMD-17 scores of the responder group and the non-responder group were significantly different and were lower than baseline.

### Cortical Thickness Between the MDD and HC Groups

**Figure 1:**
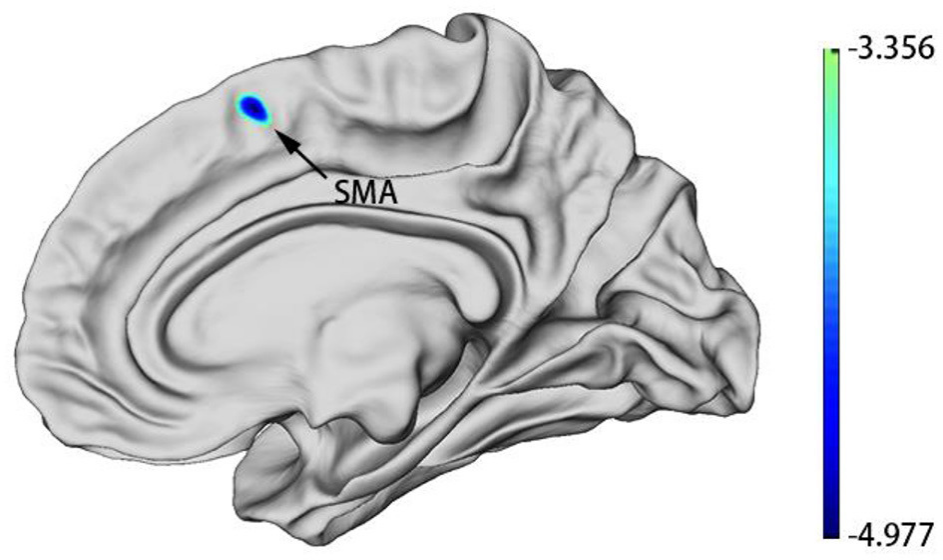
MDD group patients showed decreased cortical thickness of SMA in the right hemisphere at baseline. Blue regions represent areas of decreased cortical thickness.

Whole-brain analyses revealed that the right supplementary motor area (SMA) was thinner in MDDs when compared with HCs at baseline. All results were reported using corrected Monte Carlo permutation (Figure 1; Table 3).

**Table 3:** Difference in the cortical thickness of the right hemisphere between the MDD group and the HC group at baseline.

**Brain region**	**HCP**	**Cluster size (mm^**2**^)**	**Coordinates MNI**			**Peak intensity**
			**X**	**Y**	**Z**	
MDD < HC						
SMA	43	130	9.88	12.18	54.87	−4.9777

*Monte Carlo permutation correction was applied for each hemisphere, p < 0.025. Age, sex, education, and FD Jenkinson were regressed. HCP, human connectome project; MNI, montreal neurological institute; SMA, supplementary motor area*.

**Figure 2:**
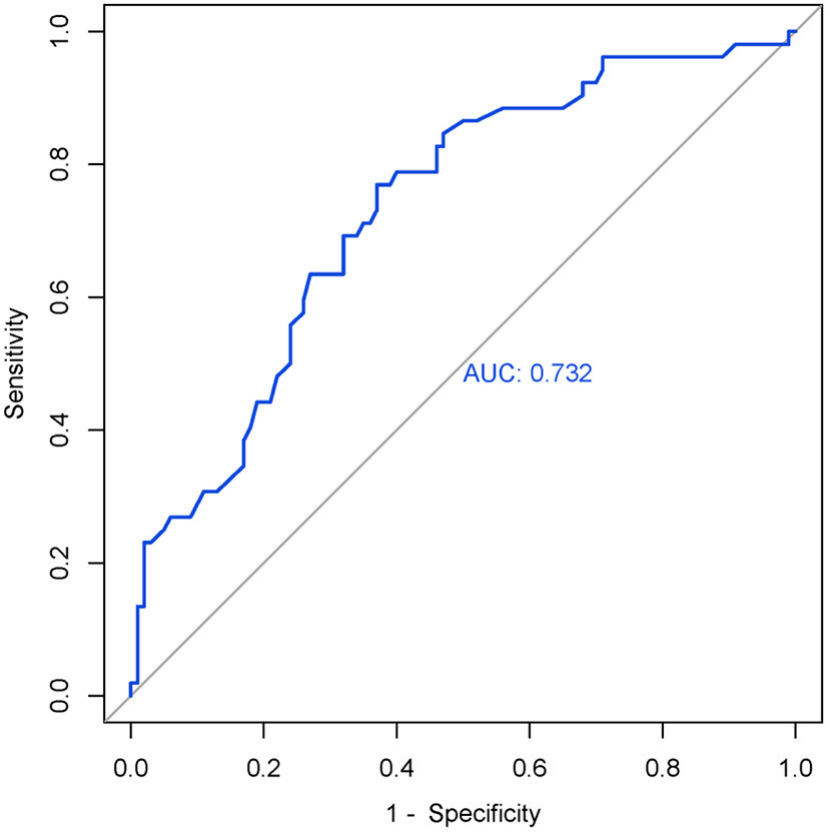
ROC curve of the right SMA's cortical thickness (0 weeks) in MDD. AUC = 0.732 (95% CI = 0.233–0.399), sensitivity = 76.9%, specificity = 63%, cutoff value = 0.399.

After we obtained the differing brain regions between the MDD and HC groups, we extracted cortical thickness values of right SMA (0 weeks) (Table 4), and we applied ROC curve for analyzing its efficacy as a diagnostic biomarker for MDD. Furthermore, we calculated ROC curve Youden index (sensitivity + specificity – 1), using the maximum value of Youden index as the basis for the optimal boundary value, which allowed us to calculate sensitivity and specificity. As shown in Figure 2, the area under the curve (AUC) of the cortical thickness at baseline in different brain areas was 0.732 [95% confidence interval (CI) = 0.233–0.399], sensitivity = 76.9%, and specificity = 63%, and the cutoff value was 0.399. The ROC curve analysis showed better results in correctly identifying MDDs than that for HCs.

**Table 4:** Cortical thickness of SMA in the right hemisphere of MDDs and HCs at baseline.

**Brain region**	**Cortical thickness (mm)**		***p***
	**MDD group** **(*n* = 100)**	**HC group** **(*n* = 52)**	
SMA	2.93 ± 0.36	3.23 ± 0.34	<0.001

*Data are mean ± standard deviation*.

### Cortical Thickness Between the Responder and Non-responder Groups at Baseline

**Figure 3:**
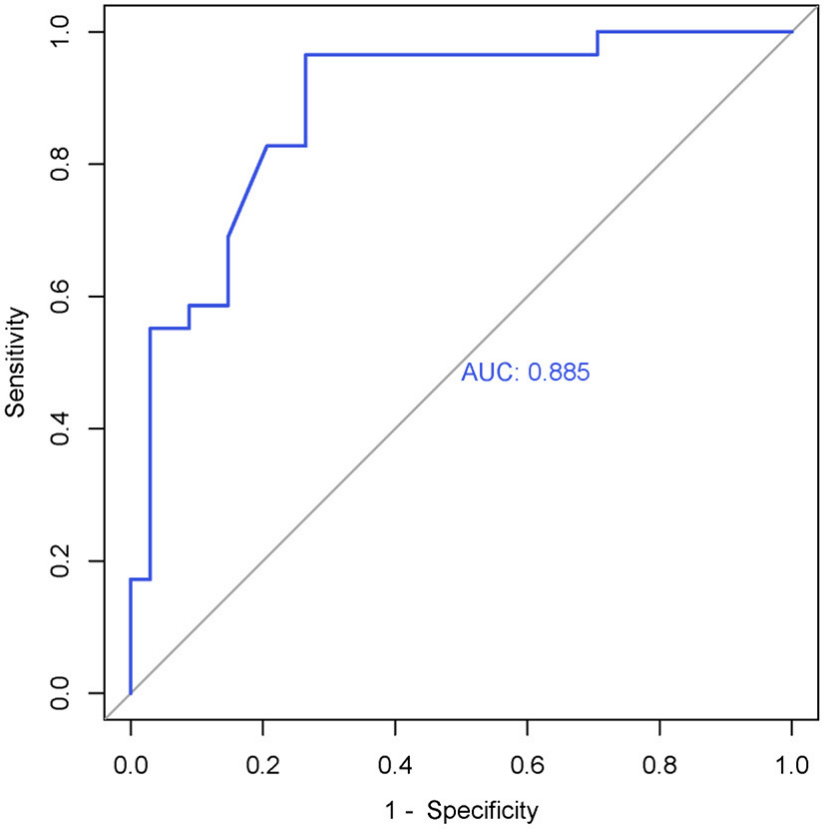
ROC curve of the right SMA's cortical thickness (0 weeks) in the responder group. AUC = 0.885 (95% CI = 0.803–0.968), sensitivity = 73.5%, specificity = 96.6%, cutoff value = 0.701.

The cortical thickness of right SMA in the responder group (*n* = 29) and non-responder group (*n* = 34) was extracted. We used an independent *t*-test to compare the cortical thickness of right SMA between the two groups at baseline. Statistic results are shown in Table 5. The cortical thickness of right SMA at baseline in the responder group was significantly thinner than that in the non-responder group at baseline. As shown in Figure 3, the AUC was 0.885 (95% CI = 0.803–0.968), sensitivity = 73.5%, and specificity = 96.6%, and the cutoff value was 0.701.

**Table 5:** Cortical thickness of SMA in the right hemisphere of the responder and non-responder groups at baseline.

**Brain region**	**Cortical thickness (mm)**		***p***
	**Responder group** **(*n* = 29)**	**Non-responder group** **(*n* = 34)**	
SMA	2.68 ± 0.26	3.14 ± 0.28	<0.001

*Data are mean ± standard deviation*.

### Correlation Between the Baseline Cortical Thickness and Clinical Symptoms

**Figure 4:**
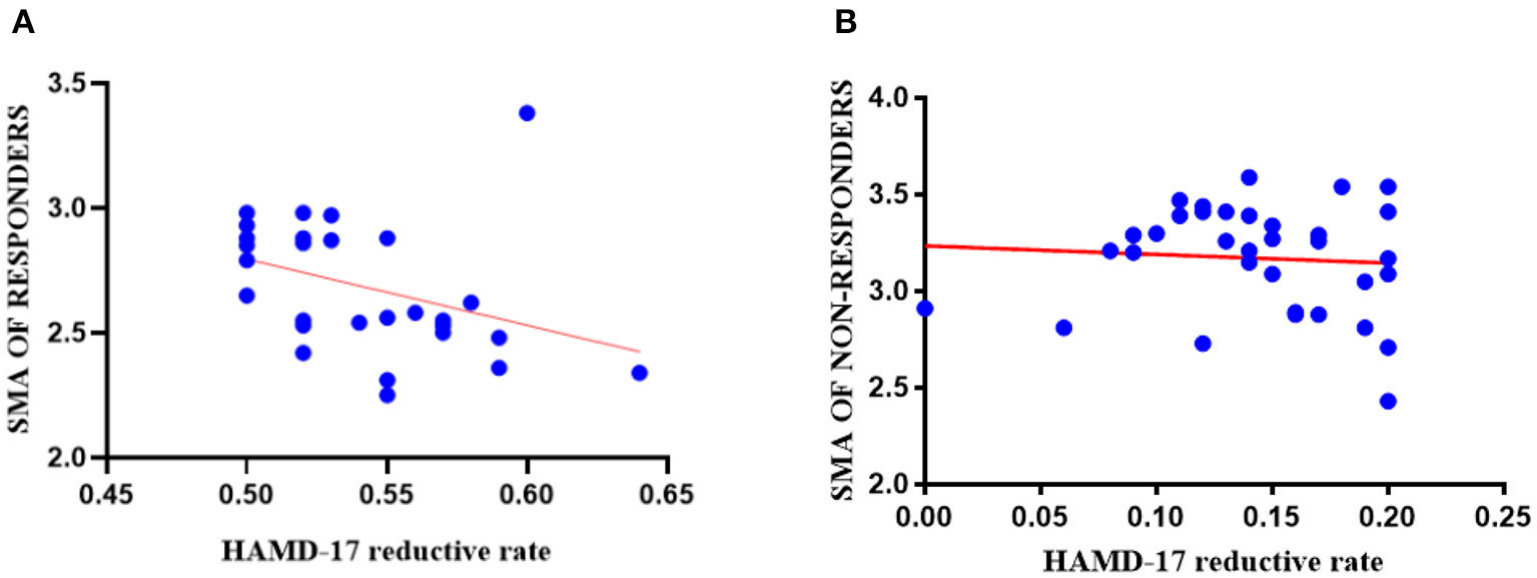
The results of correlation analyses in the responder group and the non-responder group. **(A)** The cortical thickness of right SMA (0 weeks) in the responder group had a negative correlation with 2-week HAMD-17 reduction (*r* = −0.373, *p* = 0.044); **(B)** The cortical thickness of right SMA (0 weeks) had no statistically significant correlation between the non-responder group and 2-week HAMD-17 reduction (*r* = −0.072, *p* = 0.550).

*Post-hoc* analysis, two-tailed Pearson partial correlation analysis controlling for age, gender, and education was performed for the responder group and the non-responder group separately. As shown in Figure 4, there was a negative correlation between the HAMD-17 reductive rate (2 weeks) and the cortical thickness of right SMA at baseline in the responder group (*r* = −0.373, *p* = 0.044), but there was no significant statistical difference in the correlation analysis of the non-responder group (*r* = −0.072, *p* = 0.550).

## Discussion

The main purpose of our study was to predict the treatment efficacy of SSRIs by using non-invasive sMRI, a process that allowed us to analyze the cortical thickness of MDD patients at baseline. We also analyzed different cortical thickness regions between MDDs and HCs at baseline, further examining if the brain regions have the ability to diagnose MDD. To the best of our knowledge, our study is the first study with a relatively large sample size and where MDD patients received SSRIs without other antidepressants in 2-week therapy. Findings from the present study revealed that the cortical thickness of first-episode, drug-naive patients with MDD at the baseline was decreased in the right SMA and that a significantly thinner cortical thickness in right SMA at baseline was found to be correlated with a good treatment outcome of SSRIs. Our study results suggest that the reduced cortical thickness of right SMA may function as a neuroimaging biomarker for the diagnosis of MDD and that greater decreased cortical thickness in the right SMA at baseline may be predicted with better the treatment effects of SSRIs in MDD.

As the main structural characteristic of the cerebral cortex, cortical thickness has been confirmed to be highly related to the etiology, development, and outcome of MDD. A recent study about the cortical thickness of MDD showed that the brain regions highly relevant to the pathogenesis and course of MDD are also related to the neurobiology of the antidepressant treatment efficacy (44). Supplementary motor area located in the dorsomedial frontal cortex, anterior to the primary motor cortex (45, 46), and SMA may play a key role in human volition, executive function, and the integration of affective, behavioral, and cognitive functions, the planning of complex movements. Supplementary motor area seems to be crucial for linking human cognition to action (47–49). Meanwhile, SMA is part of the foundation of voluntary capacity. Voluntary action is an essential capacity for humans, especially for adults; it is closely related to human survival, and voluntary capacity disorders are a characteristic of many mental illnesses (48). The above SMA functions are intimately linked to depressive symptoms. Previous studies about cortical thickness in MDD have focused mainly on alterations in the cingulate cortex, temporal cortex, orbitofrontal cortex, and hippocampus (29, 44, 50, 51). Although SMA was not the focus of some previous studies, the structural or functional defects of the SMA area are consistent in MDD. The region results of our study were located in the right hemisphere. The right hemisphere involves the processing of negative emotions, and a thinner cortex of the right hemisphere is associated with more serious inattention as well as current symptom severity. Furthermore, in high-risk populations, the right hemisphere's cortical thinning may increase the risk for developing a depression disorder (52, 53). Some studies confirmed that the regional cortex size of the right SMA were reduced in melancholic MDD patients and that the gray matter volume of SMA was decreased in first-episode drug-naive adult MDD subjects (54, 55). These results might be relevant to psychomotor retardation symptoms in drug-naive MDD patients, and we suppose that the structural defects of SMA may occur in the early stage of MDD or even before onset. A recent study showed that SMA can predict ECT treatment response in MDD; the prediction accuracy achieved 89% (56). Our results, in part, corroborate the conclusions reached by the aforementioned studies. However, some studies suggest that cortical volume is not mainly affected by cortical thickness (57) and that it might be related to illness duration or different treatments of the subjects in the study. This should be further analyzed in future studies. Cortical thickness reflects the density and size of neuronal cells and neurophil, and a decrease in the density and size of neurons and glial cells is related to the cortical thinning of MDD patients (24). Inconsistent with the above studies, several relevant studies reported MDD patients showed increased cortical thickness in brain regions (50, 58), which may be due to sample size, the subjects' characteristics, symptom severity, medication use, MRI acquisition, or unclear alterations in immune-inflammatory systems. As such, how cortical thinning may relate to the pathogenesis of MDD still needs further research and remains to be explored.

We also found that compared with the non-responder group, the cortical thickness of the right SMA was thinner in the responder group at baseline and was negatively correlated with HAMD-17 score improvement from baseline to the end of 2 weeks. This finding possibly suggests that the more decreased cortical thickness of right SMA at baseline, the better the outcome of treatment with SSRIs. A study showed that the reduction of energy utilization in the SMA cortical region is related to the improvement of verbal, numerical, spatial, and figural reasoning ability (49), so better performance of SMA may not be associated with better activity. A longitudinal study found that after 8 weeks of antidepressant treatment, the remitter patients with MDD (*n* = 19) at baseline had a lower thickness in the right insula and right rostral middle frontal gyrus than non-remitters (*n* = 23); moreover, following treatment, the thickening degree of the cortex in the majority of brain regions in the remitter group was greater than that in the non-remitter group. Several studies noted increased cortical thickness following the amelioration of symptoms for those with mental health disorders (59–63). Kornig et al. found that the more severe the depression symptoms, the lower cortical thickness of the right insula is; however, there was no difference in severity of symptoms between the responder and non-responder groups at baseline in our study (64). Studies about treatment-resistant depression showed SMA hypometabolism has a positive response to antidepressant treatment (56), and SMA could predict the extent of response following repeated treatments (65–67). In addition, we considered that the neurotrophic factors, such as brain-derived neurotrophic factor (BDNF), connect the treatment of depression disorder with changes in cortical thickness (68, 69). Some studies suggested that the region-specific expression of BDNF can lead to different antidepressant therapeutic effects (70, 71). These conclusions compel us to consider if the expression of BDNF in the right SMA of patients with MDD is related to the effect of SSRI treatment.

Our study also had some limitations. First, the sample size in our study was not large enough. Some patients were lost to follow-up, and we are thus incapable of conducting a longitudinal study, which is needed to identify changes in cortical thickness during the course of MDD. Second, we did not assess the degree of depression in the HC group. Therefore, we cannot control the effects of depression on cortical thickness in the baseline group comparison. Third, we did not control the types of SSRIs or the medication dose. The different SSRI types and doses may affect the outcomes. The strength of our study is that all MDD participants were first-onset, drug-naive, and without any comorbidities.

In conclusion, the present study demonstrates that lower cortical thickness of the right SMA in MDD patients at baseline may be a neuroimaging biomarker for a MDD diagnosis and that a significantly thinner cortical thickness of the right SMA at baseline may predict better SSRI treatment response. The method shows the significant prospect of cortical thickness as an indicator in the diagnosis of MDD and the identification of predictors of SSRI treatment response. In future studies, larger sample sizes and longitudinal cohort studies are needed. We hope this work will benefit clinicians with decision-making in diagnoses and in turn result in beneficial treatments for MDD patients.

## Data Availability Statement

The datasets generated for this study are available on request to the corresponding author.

## Ethics Statement

The studies involving human participants were reviewed and approved by Ethical Committee of the First Hospital of Shanxi Medical University. The patients/participants provided their written informed consent to participate in this study. Written informed consent was obtained from the individual(s) for the publication of any potentially identifiable images or data included in this article.

## Author Contributions

KZ and CY designed the experiments. PW, AZ, NS, LL, PL, HL, and YW participated in the collection and analysis of clinical data, stool samples, and blood samples of all subjects. PW analyzed data and wrote the manuscript. All authors contributed to the clinical data collection and assessment.

## Funding

This study was supported by Shanxi Scholarship Council of China (HGKY2019098) and National Natural Science Youth Fund Project (81701345).

## Conflict of Interest

The authors declare that the research was conducted in the absence of any commercial or financial relationships that could be construed as a potential conflict of interest.

## Publisher's Note

All claims expressed in this article are solely those of the authors and do not necessarily represent those of their affiliated organizations, or those of the publisher, the editors and the reviewers. Any product that may be evaluated in this article, or claim that may be made by its manufacturer, is not guaranteed or endorsed by the publisher.
